# A Robust Automated Image-Based Phenotyping Method for Rapid Vegetative Screening of Wheat Germplasm for Nitrogen Use Efficiency

**DOI:** 10.3389/fpls.2019.01372

**Published:** 2019-11-05

**Authors:** Giao N. Nguyen, Pankaj Maharjan, Lance Maphosa, Jignesh Vakani, Emily Thoday-Kennedy, Surya Kant

**Affiliations:** ^1^Agriculture Victoria, Grains Innovation Park, Horsham, VIC, Australia; ^2^Centre for Agricultural Innovation, The University of Melbourne, Melbourne, VIC, Australia

**Keywords:** high-throughput phenotyping, digital imaging, controlled environment, plant growth analysis, broken-stick model

## Abstract

Nitrogen use efficiency (NUE) in crops is generally low, with more than 60% of applied nitrogen (N) being lost to the environment, which increases production costs and affects ecosystems and human habitats. To overcome these issues, the breeding of crop varieties with improved NUE is needed, requiring efficient phenotyping methods along with molecular and genetic approaches. To develop an effective phenotypic screening method, experiments on wheat varieties under various N levels were conducted in the automated phenotyping platform at Plant Phenomics Victoria, Horsham. The results from the initial experiment showed that two relative N levels—5 mM and 20 mM, designated as low and optimum N, respectively—were ideal to screen a diverse range of wheat germplasm for NUE on the automated imaging phenotyping platform. In the second experiment, estimated plant parameters such as shoot biomass and top-view area, derived from digital images, showed high correlations with phenotypic traits such as shoot biomass and leaf area seven weeks after sowing, indicating that they could be used as surrogate measures of the latter. Plant growth analysis confirmed that the estimated plant parameters from the vegetative linear growth phase determined by the “broken-stick” model could effectively differentiate the performance of wheat varieties for NUE. Based on this study, vegetative phenotypic screens should focus on selecting wheat varieties under low N conditions, which were highly correlated with biomass and grain yield at harvest. Analysis indicated a relationship between controlled and field conditions for the same varieties, suggesting that greenhouse screens could be used to prioritise a higher value germplasm for subsequent field studies. Overall, our results showed that this phenotypic screening method is highly applicable and can be applied for the identification of N-efficient wheat germplasm at the vegetative growth phase.

## Introduction

Over the past five decades, there has been a significant increase in global food production resulting, in part, from the major contribution of substantial nitrogen (N) fertilizer application. Nevertheless, food production must be increased to sufficiently meet the projected world population of 9 billion people by 2050 (Godfray et al., 2010). However, with current agricultural practices this means that more than 240 million metric tons of additional N fertilizer would be utilized (Good et al., 2004). Approximately 110 million metric tons of synthetic N fertilizers are used annually for farming and food crop production globally (International Fertilizer Industry Association, 2013). However, nitrogen use efficiency (NUE) is generally low, with only 40% of applied N being taken up by the crop plants, while the remainder is lost to the environment resulting in increased production costs and environmental pollution (Good and Beatty, 2011; Nguyen et al., 2015), as well as affecting human health (Ahmed et al., 2017). Annually, excessive N application is estimated to cost up to €320 billion of damage to the environment in Europe (Brink et al., 2011). Proper N fertilization management practices are expected to reduce N fertilizer application while maintaining stable crop production (Good et al., 2004; Good and Beatty, 2011). To achieve this, improving NUE in crops is one of the most effective ways to ensure current crop yields can be maintained while N supply is reduced, or increasing crop yields with an optimum N input (Cormier et al., 2013).

NUE is a complex concept, but can be defined as the function of two varying components: N uptake efficiency (NUpE)—the plant’s ability to obtain N from the soil—and N utilisation efficiency (NUtE)—the plant’s ability to assimilate and remobilize absorbed N into the grain (Moll et al., 1982; Craswell and Godwin, 1984; Xu et al., 2012). In simplistic terms, NUE is determined by a plant’s ability to utilise supplied N into biomass and grain yield, and can be calculated as the ratio of biomass or grain yield to the amount of N inputs (Nguyen et al., 2016; Hawkesford, 2017). Multiple approaches have been proposed for NUE improvement in crops that include applications of advanced agronomical practices, genetic improvement through molecular breeding, and genetic engineering (Hirel et al., 2007; Hawkesford, 2014; Beatty and Good, 2018; Nguyen and Kant, 2018).

Among these, molecular breeding for N-efficient varieties is considered the most effective method to lift NUE in wheat (Cormier et al., 2016), although this approach depends on the availability of reliable and accurate molecular markers linked to N-efficient genes for marker assisted and genomic selection (Cabrera-Bosquet et al., 2012; Garnett et al., 2015; Han et al., 2015). However, molecular breeding for N-efficient varieties is still a daunting task, given that NUE is a polygenic trait with complex interactions, and associated genes are heavily influenced by environmental conditions such as varying soil N, soil type, rainfall pattern and soil water availability (Cormier et al., 2013; Lammerts Van Bueren and Struik, 2017; Nguyen et al., 2017). A large volume of high-quality phenotypic data is needed to dissect NUE’s genetic influences into smaller manageable and measurable components, and to derive reliable and accurate molecular markers or genomic estimated breeding values (Araus and Cairns, 2014; Nadeem et al., 2018). Thus, molecular breeding goals for N-efficient varieties rely heavily on the deployment of effective phenotyping methods (Araus and Kefauver, 2018; Araus et al., 2018). However, the absence of a robust, high-throughput, and reliable phenotyping method that is powerful enough to break down genetic components is currently limiting breeders’ efforts to make a breakthrough in the genetic improvement of NUE traits (Nguyen and Kant, 2018). Effective screening methods for identifying N-efficient germplasm that performs consistently in the greenhouse and field conditions are required to facilitate breeding outcomes (Garnett et al., 2015; Nguyen and Kant, 2018). High-throughput phenotyping methods which can effectively differentiate the performance of germplasm at early growth stages and predict their performance at harvest are urgently required (Sharma and Bali, 2018).

Over the last two decades, proximal sensing technology has become one of the most promising high-throughput phenotyping approaches that can provide key non-destructive support in measuring performance and predicting crop yield in controlled and field environments (Fiorani and Schurr, 2013; Araus and Cairns, 2014; Araus and Kefauver, 2018). This technology was fundamentally developed on the principle that the light reflectance from the interaction between the natural light spectrum with plant components could provide accurate information on the morphological and physiological status of plants (Homolová et al., 2013; Fahlgren et al., 2015). The light reflectance captured by specially designed optical instruments can then be used to generate vegetation indices (VIs) and digital plant objects. Once validated, these derived VIs or digital plant objects can be used as proxies of various plant traits, such as shoot biomass, leaf area or N content, to compare the performance of individual varieties (Nguyen and Kant, 2018). For instance, the most common vegetation index i.e. normalised difference vegetation index (NDVI), is often used to assess a plant response to varying N supplies (Nguyen et al., 2016). Moreover, the application of optical devices such as Red-Green-Blue (RGB) digital cameras has also been used for measuring crop growth, phenology and yield components, as well as the development of VIs (Casadesús et al., 2007; Casadesús and Villegas, 2014; Nguyen et al., 2018). Unlike conventional spectral indices, RGB indices are not affected at long wavelengths by elements such as crop architecture and soil cover, and they were shown to outperform the conventional spectral indices in measuring crop growth and N use to some extent (Araus and Kefauver, 2018). There have been a few reports on the application of automated RGB imaging platforms to study phenotypic responses of grass species (Poiré et al., 2014), and sorghum (Neilson et al., 2015; Berry et al., 2018) to N fertilizer under controlled environments. Recently, ground and aerial based RGB imaging has been successfully used to study NUE in wheat and maize under field conditions (Prey et al., 2018; Buchaillot et al., 2019). However, a robust method using imaging technology for screening of crop germplasm for NUE under controlled conditions is still to be reported. To the best of our knowledge, this is the first study describing a vegetative phenotypic screening method for NUE improvement in wheat using automated imaging phenotyping technology under controlled environments.

The aim of this work was to develop a high-throughput and high-resolution phenotyping protocol that can effectively screen wheat varieties at the vegetative growth phase for NUE improvement in controlled environments, and then to compare the performance of wheat varieties under controlled and field conditions with respect to NUE. The perspectives of applying sensing technologies for phenotypic screens of wheat germplasm for NUE under field conditions are also discussed.

## Materials and Methods

### Plant Materials and Growth Conditions

**Figure 1:**
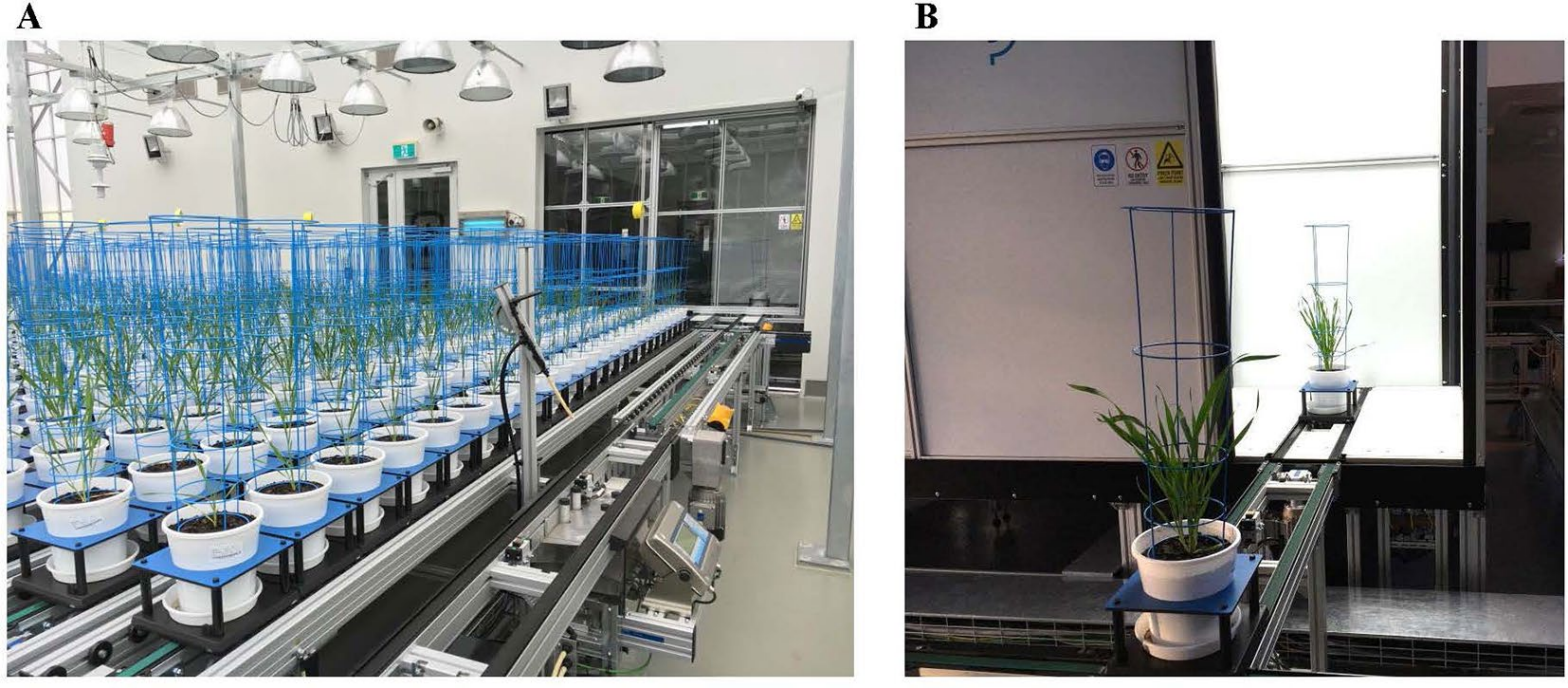
The automated system in Plant Phenomics Victoria, Horsham. **(A)** Pots laid out on conveyor system with a watering and weighing station. **(B)** Plants moving to imaging booth containing a side and a top RGB camera for image acquisition.

Fifteen genotypically diverse wheat (*Triticum aestivum* L.) varieties used in our previous field trial (Nguyen et al., 2016), where they were shown to be differentially responsive to N, were studied in two separate experiments at Plant Phenomics Victoria, Horsham, described in detail by Nguyen et al. (2018). Briefly, the automated phenotyping platform consists of a conveyor belt system, a watering and weighing station (**Figure 1A**), and an imaging chamber with a Scannalyzer 3D imaging system (LemnaTec GmbH, Aachen, Germany; **Figure 1B**).

In the first experiment, two varieties, Bobwhite and Chara, were used for the identification of appropriate low and optimal N levels for further screens. White plastic pots (200 mm diameter x 190 mm deep, Garden City Plastics Pty Ltd, Victoria, Australia) were filled with 3.5 litres of cereal standard soil mix without added fertilizers (Biogro, South Australia, Australia). To ensure that each pot was filled with an equal amount of soil, pots were weighed prior to sowing. Three seeds were sown per pot on rolling benches and thinned to one plant at 3 leaf stage (∼ 2 weeks old). To avoid water leaking, pots were placed on white saucers for the duration of the experiment. On a weekly or fortnightly basis, between 100 ml and 200 ml of nutrient solution, components listed in **Supplementary Table 1**, was supplied depending on the crop growth stages (vegetative or reproductive). Ferrous fertilizer (Fe^3+^) was supplied as Librel^®^ Fe-LO (CW Pacific Specialties Pty Ltd, NSW, Australia) and phosphorus fertilizer (PO_4_^-3^) was supplied as a phosphate buffer with pH 6. Six relative N levels using KNO_3_ as the sole N source were applied; 2 mM, 5 mM, 10 mM, 15 mM, 20 mM, and 25 mM N. Water was supplied adequately and equally among pots to keep plants growing healthily. Due to some greenhouse conditions, we could not continue the experiment until crop maturity. However, the crop growth and canopy development were carefully observed and used as guidance for the second experiment.

In the second experiment, 15 wheat varieties were screened for their responsiveness to two N levels, 5 mM and 20 mM, designated as low and optimum N. Under our observation, the total amount of N supplied for these two levels was equivalent to 147 mg and 588 mg N per pot, respectively, which were similar to the N levels supplied in the previous study by Malik et al. (2016). Pot preparation and plant growth management were conducted similarly to the first experiment at the Plant Phenomics Victoria, Horsham. Three weeks after sowing, pots were loaded and laid out on the conveyor belt system in a split-plot design with 15 replicates per N treatment, where N was the main treatment and variety was the sub-treatment. The growth conditions in the greenhouse were 24°C during the day and 16°C during the night with a 12 h photoperiod. To keep plants upright for imaging, a cage was placed into each pot, which was painted blue so that its images could easily be segmented and removed as background noise during image analysis.

Wheat plants at both N treatments were divided into two sets. The first set of plants was harvested at 49 days after sowing (DAS) for the vegetative growth evaluation. The second set continued to grow until maturity and was harvested for grain yield and yield attributes assessment. Heading date was recorded as the day the first head in each pot completely emerged as described previously by Guedira et al. (2016). Physiological maturity date was recorded as the day when the lower glumes of spikes completely lost all the green colour.

### RGB Image Acquisition and Analysis

**Figure 2:**
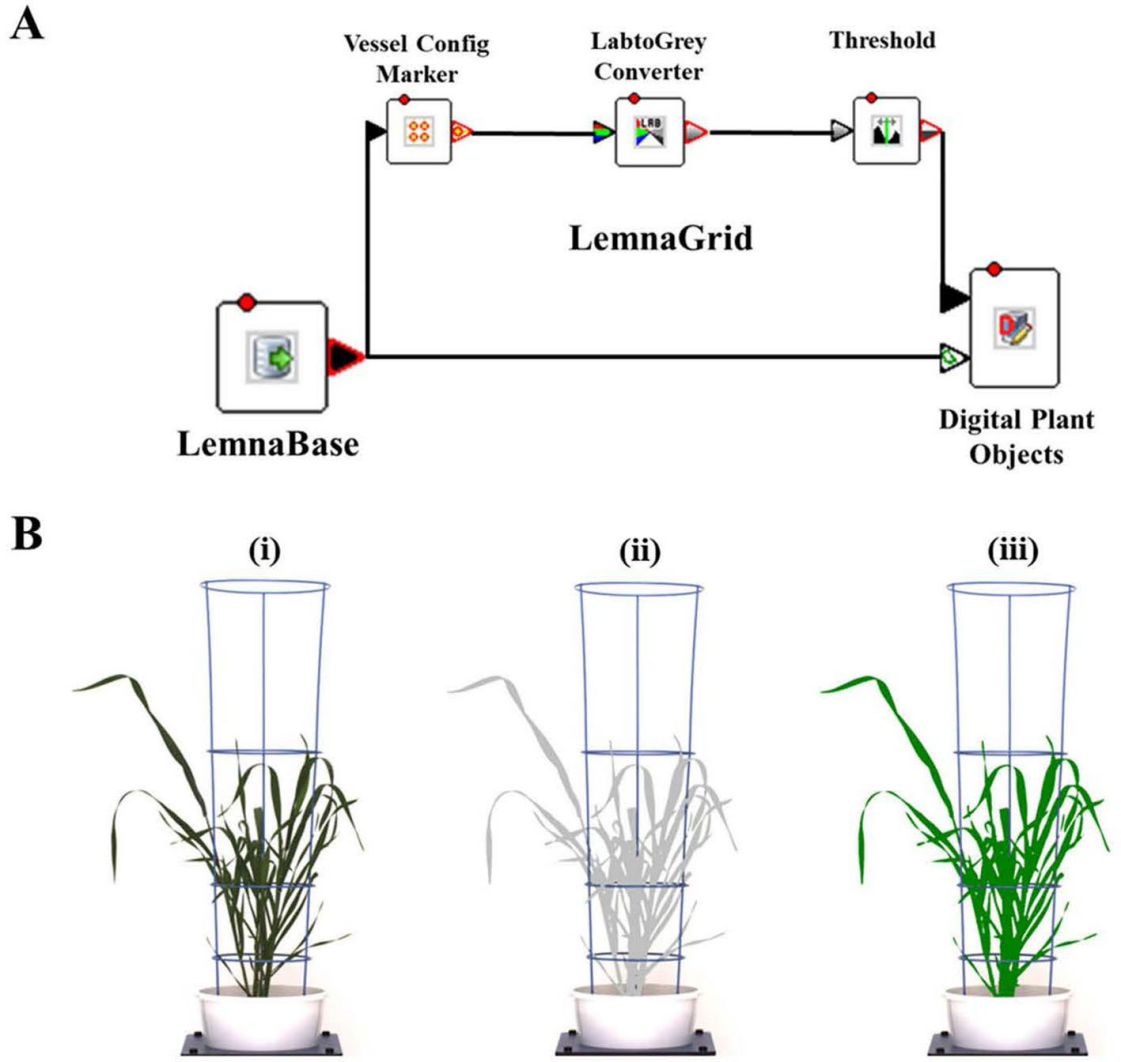
Image acquisition and analysis by the automated plant phenotyping system, Plant Phenomics Victoria, Horsham. Panel **(A)**, a simplified image processing algorithm comprising key steps and devices of LemnaGrid software. Panel **(B)**, i) a raw side-view image of a wheat plant cv. Bobwhite at 49 DAS; ii) the color classification and identification of corresponding object (the grey plant); iii) the processed image object (the bright green plant). Estimated shoot biomass is the pixel sum of highlighted green objects in processed images.

Digital image capture and analysis were implemented following the procedure described previously by Nguyen et al. (2018). In brief, after loading plants on the automated system, they were imaged twice a week by the Scannalyzer 3D plant-to-sensor imaging unit which consists of two 28.8 megapixel RGB cameras (a side and a top camera), and model Prosilica GT6600C (Allied Vision Technologies, Stadtroda, Germany) (**Figure 1B**). Three colour images were acquired from 3 sides of the plant after consecutive rotations of 0, 120, 240 degrees, and a top-view image of the plant was also acquired. Images taken were automatically recorded in the database server which is managed by LemnaBase software (LemnaTec GmbH, Aachen, Germany). **Figure 2A** illustrates a simplified image processing algorithm, containing key steps and devices of LemnaGrid software (LemnaTec GmbH, Aachen, Germany). To analyse the images, the region of interest consisting of whole plant parts in the raw images (**Figure 2B**, i) was separated from the background by Vessel Cofig Marker device. In subsequent steps, the noise was removed from the region of interest and purified by LabtoGrey Converter and Threshold devices (**Figure 2A**) and finally the digital plant objects were determined (**Figure 2B**, ii). This object was used to estimate morphological and physiological traits of the plant (the bright green objects, **Figure 2B**, iii). **Table 2** lists traits measured by digital plant objects and conventionally destructive methods.

### Destructive Phenotyping

The first set of plants was destructively harvested at 49 DAS by cutting plants above the soil level in the pots. Whole plants were immediately weighed to determine fresh biomass (MB) per pot. All leaves from the plant were then detached from stems and used to determine leaf area (LA) using a Portable Area Meter, model LI-3050A (LI-COR Inc, Lincoln, Nebraska, USA).

The second set of plants was harvested at physiological maturity to determine yield and yield attributes as described in **Table 1**. All above ground parts of the plant were removed from pots and oven-dried at 65°C for 5 days. After the measurement of total dry biomass (DW), the spikes were separated from stems and counted to determine number of spikes per pot (SN), as well as number of grains per spike (GN), total grain yield (GY) and 1000-grain weight (1000-GW).

**Table 1:** Wheat traits measured by digital RGB imaging and destructive methods.

Traits	Abbreviation	Unit	Description
Estimated shoot area	EB	kilopixel (kPix)	The pixel sums of three side-views and top-view image of the plant
Top-view area	TVA	kPix	The pixel sums of the top-view image
Measured shoot bimass	MB	gram (g)	Destructive biomass harvest at 49 DAS
Measured leaf area	LA	cm^2^	Total leaf area per plant per pot
1000-grain weight	1000-GW	gram (g)	Manually count and weigh 1000 grains
Number of spikes	SN	spike	Manually count the number of spikes per pot
Number of grain per spike	GN	grain	Manually count the number of grain per spike
Dry biomass	DW	gram (g)	Determined by manually weighing total dry biomass per pot
Grain yield	GY	gram (g)	Determined by manually weighing total seed yield per pot

### Water Soluble Carbohydrate Assay

Water soluble carbohydrate (WSC) concentration of plant shoots was determined by near-infrared reflectance (NIR) spectroscopy. The ground plant samples were measured using FOSS XDS Rapid Content Analyser (FOSS, Hillerød, Denmark). The WSC composition was predicted using the WINISI 4 NIR calibration with standard error of prediction of 1% and R^2^ of 0.98. The reference WSC method used for validating NIR data was adopted from Maharjan et al. (2018).

### Shoot and Grain N Concentration Measurement

The procedure for shoot and grain N concentration measurement was described previously by Nguyen et al. (2016). Briefly, a subset of samples was randomly collected from shoot dry biomass or grain samples and ground to fine powder by a grinder (Cyclotec; Foss, Hillerød, Denmark). Total N concentration in the shoot and grain samples were determined by NIR spectroscopy (Foss XDS Rapid Content Analyser) (AACC method 39-25) and calculated on a dry weight basis.

### Nitrogen Use Efficiency

The NUE of wheat plant biomass and grain per pot was calculated according to the formula adapted from Crasswell and Godwin (1984) with modifications.

(1) $${\text{NUE}}_{\text{b}}=\;W_{\text{biomass}}/W_{\text{N inputs}}$$

(2) $${\text{NUE}}_{\text{g}}=\;W_{\text{grain}}/W_{\text{N inputs}}$$

where NUE_b_ (1) and NUE_g_ (2) are the nitrogen use efficiency of wheat plants per pot in regard to biomass and grain yield, respectively; W_biomass_ and W_grain_ are the weight (grams) of plant biomass and grain yield per pot at harvest, respectively; W_N inputs_ is the amount (grams) of nitrogen inputs.

### Comparison of the Performance of Wheat Varieties Under Greenhouse and Field Conditions

To compare the performance of 15 wheat varieties grown under greenhouse and field conditions, we utilized the published data set from our previous field trial (Nguyen et al., 2016). Harvest plant dry biomass and grain yield from this study were compared with the performance of the same wheat genotypes grown in the field trial.

### Plant Growth Model and Statistical Analysis

Imaging-derived and manually measured data were initially checked for outliers by using GENSTAT statistical software version 18.0 statistical software (VSN International Ltd, Hemel Hempstead, UK). Two-way analysis of variance (ANOVA) was performed to determine varietal effects by using the same software. The procedure for the selection of the best fit nonlinear regression plant growth model based on the estimated biomass and statistical analyses was adopted from Nguyen et al. (2018). In brief, the biomass accumulation of wheat plants over the growth period follows a sigmoidal growth pattern (Malhi et al., 2006; Archontoulis and Miguez, 2015) and the “broken-stick” statistical model fitting two straight lines using GENSTAT was used to identify the linear growth phase of wheat plants. Linear regressions and Pearson’s correlation coefficient (*r*) were used to determine the correlations between estimated and measured plant traits by using R statistical software (version R-3.5.0) (R Core Team, 2017).

## Results

### Wheat Varietal Response to Various N Supplies

**Figure 3:**
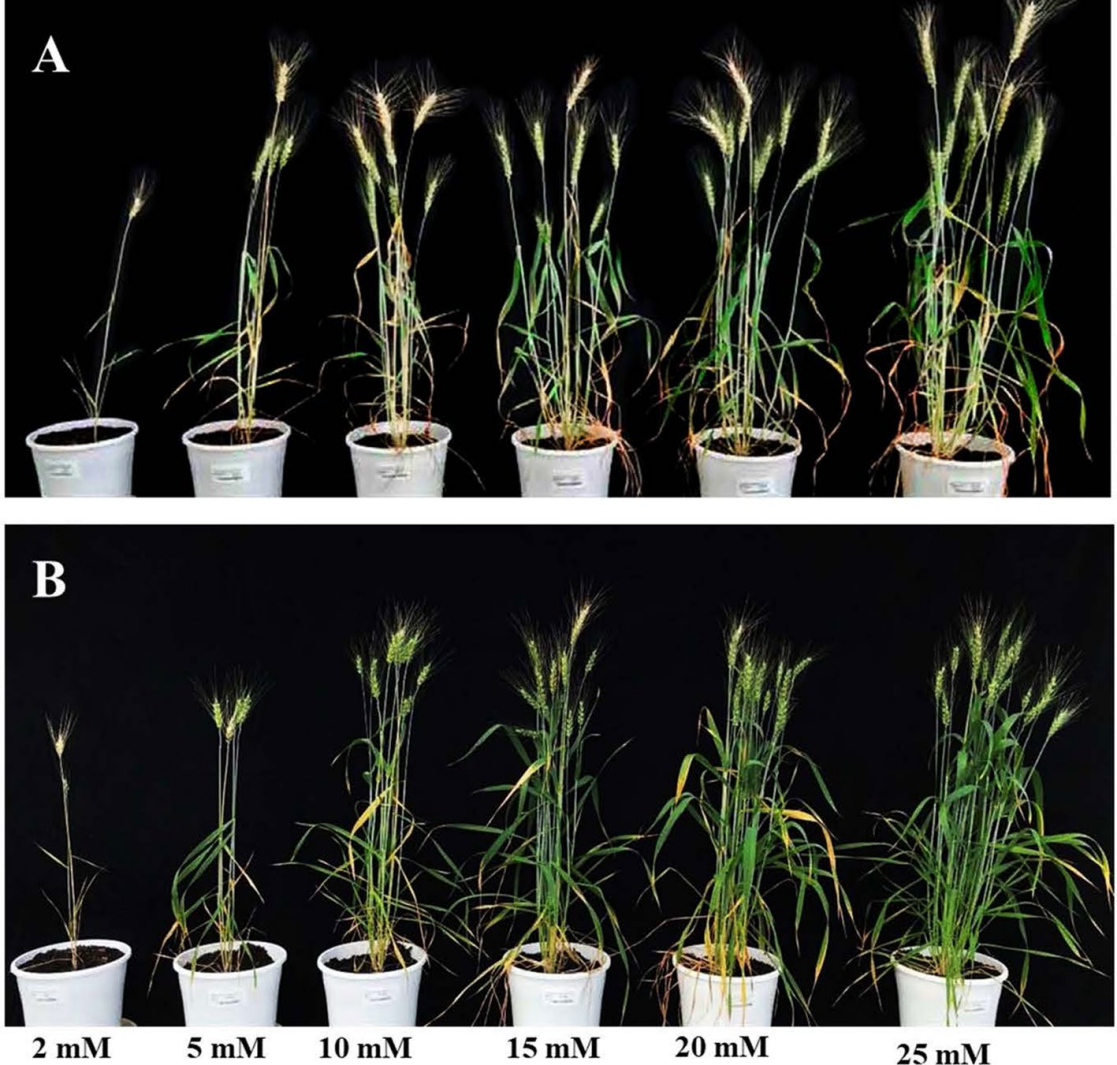
Wheat varieties Bobwhite **(A)** and Chara **(B)** grown under six levels N levels: 2 mM, 5 mM, 10 mM, 15 mM, 20 mM, and 25 mM. Photos taken at 90 DAS.

The initial experiment screened six N concentrations, 2 mM; 5 mM; 10 mM; 15 mM; 20 mM and 25 mM on two bread wheat cultivars, Bobwhite and Chara. Overall, data showed that increased N concentrations resulted in a larger canopy and higher biomass accumulation (**Figure 3**). Plants did not grow well at 2 mM N, whereas they appeared over grown at 25 mM N with very large canopies. Leaf overlap due to large canopies can reduce the correlation of the digital image to actual biomass, especially when leaf area index > 3 (Serrano et al., 2000). Therefore, the two N concentrations, 5 mM and 20 mM were chosen and designated as low and optimum N levels, for subsequent screens of wheat genotypes for NUE traits.

In the second experiment, 15 genetically diverse wheat cultivars including Bobwhite and Chara were grown under the low and optimum N levels. Overall, all varieties showed positive responses to the increased N supply, resulting in longer growth duration, higher DW, GY, shoot and grain N concentration (**Supplementary Figure 1** and **Table 2**). Data showed that wheat varieties responded differentially to the supplied N leading to a significant N and variety interaction (**Table 2**). Varieties such as Yitpi, Chara and Alsen had high DW accumulation, in contrast to Westonia, Kennedy and Drysdale, which accumulated less DW at both N levels (**Table 2**). Grain yield showed a highly positive association with DW accumulation at both N levels (**Table 2**). Greater DW accumulators such as Yitpi, Chara, and Alsen also had higher GY than Westonia, Kennedy and Drysdale (**Table 2**). However, the shoot and grain N concentrations of wheat varieties showed a highly negative trend with DW accumulation, GY and WSC at both N levels. Varieties with the lowest DW, GY, and WSC such as Kennedy and Drysdale had shoot and grain N concentration higher than Yitpi, Chara and Gladius at both N levels (**Table 2**). WSC concentrations of plants at maturity were higher under optimum N than low N for all the varieties (**Table 2**). Interestingly, WSC concentrations corresponded more with DW and GY at low N than optimum N (**Table 2**). On average, wheat varieties at optimum N level had higher SN, but slightly lower harvest index than those at the low N level (**Table 3**). However, the 1000-GW and GN were not changed significantly (**Table 3**).

**Table 2:** Shoot dry biomass, grain yield, shoot water soluble carbohydrates, shoot and grain N concentration of wheat varieties grown under low and optimum N treatments at maturity. Varieties are ranked in descending order of DW at low N level. In a column: dark green cells, the highest values; dark red cells, the lowest values; DW, plant dry biomass; GY, grain yield; WSC, shoot water soluble carbohydrate; s.e.d., standard error difference of the means.

Variety	DW (g pot^-1^)				GY (g pot-1)				WSC (%)				Shoot N (%)				Grain N (%)			
	Low N		Optimum N		Low N		Optimum N		Low N		Optimum N		Low N		Optimum N		Low N		Optimum N	
Yitpi	19.21		77.67		7.34		27.77		8.52		10.52		0.40		0.44		1.65		2.26	
Excalibur	15.74		57.95		7.74		24.62		5.55		8.77		0.36		0.70		1.94		2.86	
Chara	13.43		61.13		6.52		29.05		6.18		8.32		0.31		0.56		2.11		2.51	
Pastor	13.34		60.53		6.46		28.13		3.03		6.58		0.33		0.57		1.90		2.51	
Bobwhite	12.66		50.02		6.31		24.54		4.30		8.05		0.28		0.74		2.07		2.61	
Alsen	12.59		61.37		6.13		28.23		5.18		9.33		0.35		0.49		2.30		2.73	
Gladius	12.30		58.60		6.13		28.26		6.00		11.32		0.31		0.50		2.08		2.42	
Wyalkatchem	12.08		48.71		5.95		22.30		5.83		8.90		0.48		0.71		1.98		2.68	
RAC875	12.06		56.21		5.59		24.84		6.93		12.83		0.31		0.61		2.28		2.68	
Kukri	11.99		54.14		6.49		25.35		5.10		10.12		0.47		0.66		2.13		2.72	
Baxter	11.21		50.98		5.32		23.91		3.93		5.63		0.41		0.86		2.17		2.76	
Volcani DDI	10.53		45.16		4.95		20.69		4.35		10.45		0.29		0.55		2.45		3.17	
Westonia	10.52		49.45		5.72		23.99		4.15		8.08		0.33		0.57		2.11		2.74	
Kennedy	8.70		41.33		4.79		21.97		3.40		5.48		0.33		0.76		2.32		2.92	
Drysdale	8.26		38.78		4.54		18.87		2.97		7.00		0.34		0.90		2.31		2.78	
ANOVA	N	V		N x V	N	V		N x V	N	V		N x V	N	V		N x V	N	V		N x V
s.e.d.	0.54	1.03		1.50	0.187	0.733		1.018	0.194	0.675		0.942	0.014	0.047		0.066	0.005	0.03		0.042
p	<0.001	<0.001		<0.001	<0.001	< 0.001		<0.001	<0.001	<0.001		0.02	< 0.001	< 0.001		<0.001	<0.001	<0.001		<0.001
l.s.d. (*P* = 0.05)	2.903	3.006			2.076	2.038			0.954	0.942			0.13	0.13			0.084	0.086		

**Table 3:** Mean value of yield components of 15 wheat varieties. GW, grain weight; SN, number of spikes per pot; GN, number of grains per spike; CV, coefficient of variation; n.s., not significant difference (*p* > 0.05).

Component	Low N	Optimum N	l.s.d. (*p* = 0.05)	CV%	*p*
1000-GW (g)	41.35	44.26	3.11	9.7	ns
SN	4.21	15.38	1.83	25	< 0.001
GN	34.8	37.88	3.84	14.1	ns
Harvest index	49.41	46.36	3.02	8.4	0.05

**Figure 4:**
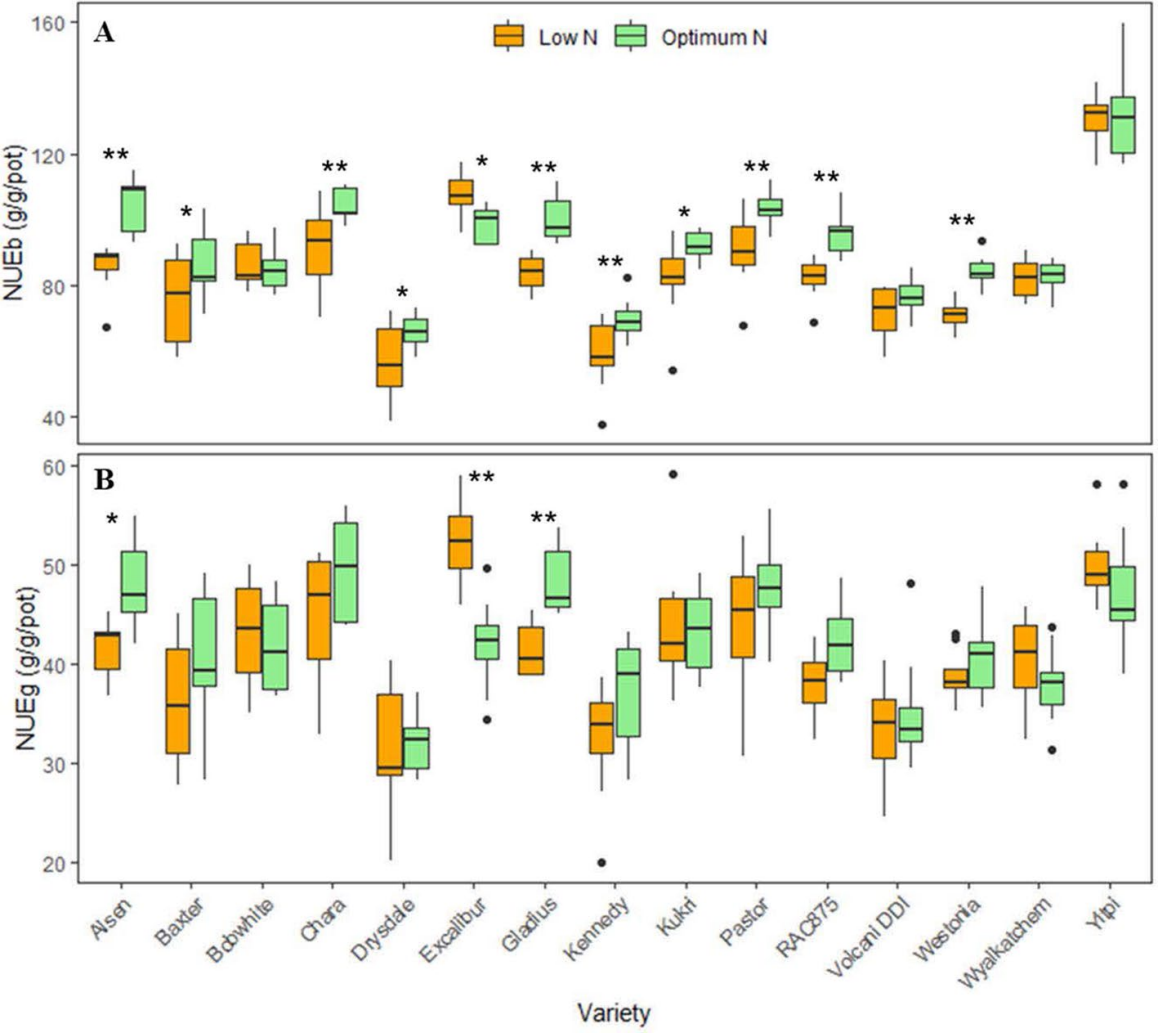
Boxplots of nitrogen use efficiencies (NUEs) of 15 wheat varieties at low and optimum N levels. **(A)** NUE_b_ is nitrogen use efficiency calculated by harvested biomass (equation 1); **(B)** NUE_g_ is nitrogen use efficiency calculated by grain yield (equation 2). The asterisks indicate the statistically significant levels of ANOVA, comparing the NUE of a variety within N levels (* p ≤ 0.05; ** p ≤ 0.01).

The boxplots showed significant variations in NUE of biomass (NUEb) and grain (NUEg) per pot between varieties and N levels, confirming a significant interaction between these two factors (**Figure 4**). Varieties such as Alsen, Chara and Yitpi had high NUEs compared to Drysdale, Kennedy and Volcani DDI. Within N levels, 11 wheat varieties had significant variations in NUEb (**Figure 4A**), while only 3 varieties i.e. Alsen, Gladius and Excalibur were significantly different in NUEg (**Figure 4B**). This suggests that N treatments resulted in a more stable NUE for grain than biomass.

### Validation of Imaging Phenotyping

**Figure 5:**
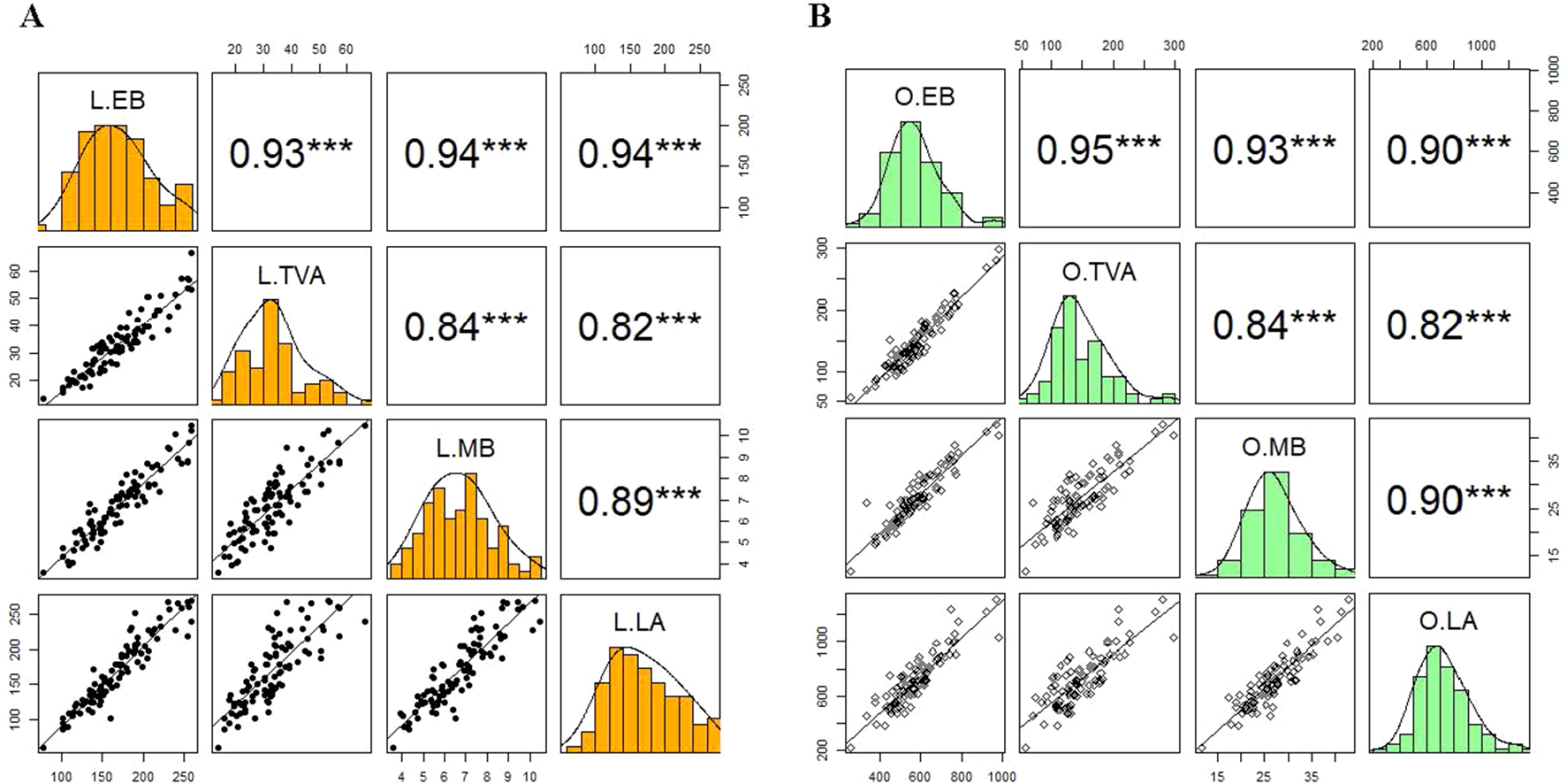
Validation of the relationships between estimated and measured NUE traits of 15 wheat varieties. Panels **A**) and **B**) represent the correlation between estimated biomass and top-view area with measured fresh shoot biomass and leaf area collected at 49 DAS at low and optimum N supplies, respectively. In each panel, the coloured windows are the histograms of individual traits. The windows above and below the diagonal of the coloured windows are Pearson’s correlation coefficients (r) and bivariate scatter plots with trend lines, respectively. L, low N; O, optimum N. EB, estimated biomass; TVA, top-view area, MB, measured biomass; LA, leaf area. The asterisks are the statistically significant levels (*** p ≤ 0.001). Sample number = 90.

To validate the application of the image-based phenotyping technology used to study the responses of wheat varieties to N supplies, we determined the association between the morphological and physiological parameters of 15 wheat varieties derived from digital imaging and conventionally destructive sampling methods (**Figure 5**). Manually harvested samples were collected at 49 DAS and used to measure MB and LA. The EB and TVA were derived from digital RGB images collected the night before the destructive harvest. Results from the correlation analysis at low N level showed that EB and TVA were highly correlated with important NUE traits such as measured fresh MB and LA for all 15 wheat varieties with statistically significant coefficients of 0.94 and 0.82, respectively (**Figure 5A**). Likewise, the correlation analysis at optimum N level showed similar results; EB and TVA were also highly correlated with these NUE traits with correlation coefficients of 0.93 and 0.90, respectively (**Figure 5B**). The results also showed that there were intercorrelations within estimated and measured NUE traits at both N levels. For instance, EB was highly correlated with TVA and MB was highly correlated with LA (r ≥ 0.89, **Figure 5**). Similarly, EB was highly correlated with LA and TVA was highly correlated with MB (r ≥ 0.84, **Figure 5**). This suggests that these traits can be used interchangeably for an effective NUE assessment.

### Association Between Vegetative Performance and Harvest of Wheat Varieties

**Figure 6:**
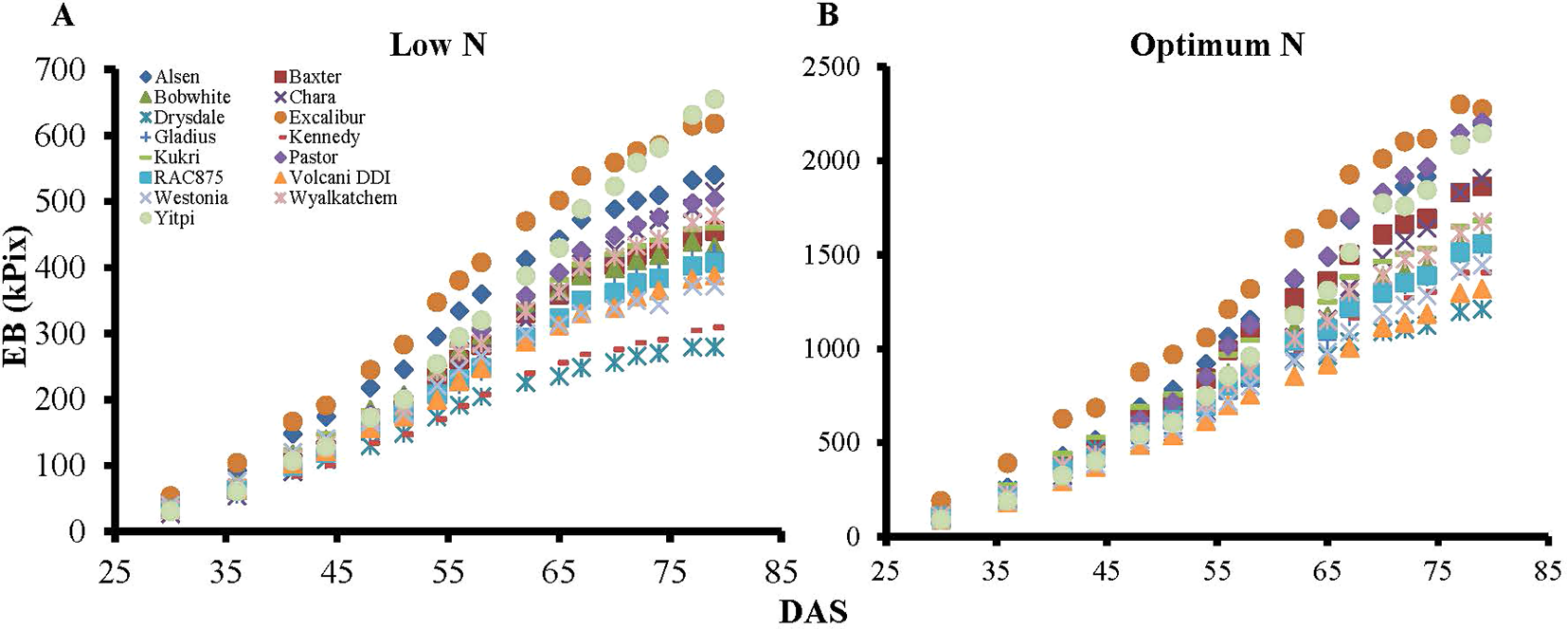
Dynamic growth of wheat genotypes under low and optimum N levels. Graph **A**) and **B**) represent biomass accumulation over the growth period for 15 wheat genotypes at low and optimum N level, respectively. EB, estimated biomass; DAS, days after sowing. Specific marker shapes and colors indicate particular genotypes.

Biomass accumulation of wheat and other grain crops over the growth period follow a sigmoidal growth pattern (Malhi et al., 2006; Archontoulis and Miguez, 2015; Nguyen et al., 2018). In the current study, our data showed that biomass accumulation (as indicated by EB) of wheat varieties under both N levels followed a sigmoidal growth pattern (**Figures 6A, B**). We determined the breakpoints where varieties commence or complete their linear growth phase using the “broken-stick” model (**Table 4**). Breakpoints are the reference points where each linear regression was “broken”, or the slope changed, given as X, Y coordinates, with X being the DAS and Y being the EB. The broken-stick model fitted well with the EB of wheat varieties, as indicated by high values of adjusted coefficients of determination (adjusted R^2^ > 0.99; **Table 4**). Regression slopes before (slope 1) and after (slope 2) the breakpoint indicate the commencement or completion of the linear growth phase, while the exact values can indicate the number of days spent in either the linear or lag growth phases during the imaging period (**Table 4**). At the low N level, ten wheat varieties, i.e. Alsen, Baxter, Bobwhite, Drysdale, Excalibur, Kennedy, Kukri, RAC875, Volcani DDI and Westonia, commenced their linear growth phase early, with the breakpoint identified in **Table 4** being at the end of linear growth rather than the start, seen by a higher slope 1 and lower slope 2. Out of these, Drysdale completed its linear growth phase the earliest at 66.3 DAS (**Table 4**). Meanwhile, the remaining five varieties commenced their linear growth phase later, with 48.7 DAS being the latest time point when one of the varieties Yitpi commenced linear growth (**Table 4**). Thus, the period between 48–66 DAS is the duration when all wheat varieties were in their linear growth phase under low N (**Table 4**). At the optimum N level, all varieties commenced their linear growth phase later between 36.1–52.4 DAS, compared to the same varieties at the low N level. However, like the results seen at low N level, Drysdale once again completed its linear growth phase earlier than all other varieties, at 62 DAS (**Table 4**). Therefore, the period between 52–62 DAS is when all wheat varieties were in their linear growth phase under optimum N (**Table 4**).

**Table 4:** Regression parameters as determined by the split-line linear regression model of 15 wheat genotypes*. X is DAS and Y is EB, which represent the coordinates of the breakpoint where the linear regression was split or broken; slope 1 and slope 2, slopes of the regression before and after the breakpoint, respectively.

Variety	Low N				Optimum N			
	Breakpoint X	Breakpoint Y	Slope 1	Slope 2	Breakpoint X	Breakpoint Y	Slope 1	Slope 2
Alsen	68.7	480.8	11.9	5.9	46.2	570.9	28.1	49.8
Baxter	71.2	413.4	10.0	5.4	43.6	454.0	26.0	41.8
Bobwhite	68.1	393.6	10.0	4.1	37.8	242.0	18.1	33.8
Chara	45.1	113.7	6.0	12.3	52.4	578.7	22.1	50.2
Drysdale	66.3	247.4	6.0	2.8	62.0	939.1	25.9	16.5
Excalibur	69.1	554.5	13.6	6.9	52.3	1024.0	37.6	53.7
Gladius	42.5	97.3	5.6	9.5	49.9	514.5	22.3	36.5
Kennedy	67.8	268.9	6.7	3.7	37.7	241.2	17.6	30.4
Kukri	69.3	419.1	10.4	4.6	36.1	273.0	22.6	34.3
Pastor	38.2	77.3	5.4	11.2	46.4	501.0	24.8	54.2
RAC875	71.8	374.5	8.8	4.8	45.7	457.3	22.8	33.8
Volcani DDI	68.6	384.4	8.3	5.4	39.2	232.7	15.5	27.6
Westonia	66.5	326.1	8.1	3.7	45.5	408.1	21.2	31.2
Wyalkatchem	38.6	81.6	5.7	10.4	51.0	619.7	24.7	39.1
Yitpi	48.7	170.4	7.9	16.4	51.9	620.7	25.5	57.4

*Adjusted R^2^ > 99%.

To validate whether EB during the linear growth phase can be used to evaluate the performance of wheat varieties for improved NUE, we compared the EB of the second plant set captured at 54 DAS against fresh shoot biomass and leaf area of the first plant set harvested 49 DAS for all wheat varieties (**Table 5**). The fifteen wheat varieties are ranked in descending order of MB accumulation at the low N level, where the dark green cells denote the higher values and in contrast the dark red cells represent the lower values (**Table 5**). Under low N, varieties such as Bobwhite and Yitpi accumulated higher MB in contrast to Drysdale and Volcani DDI (**Table 5**). However, Pastor and Alsen showed a stronger response to optimum N. Interestingly, Excalibur showed strong responses to N at both levels. Data also showed that EB and TVA were well correlated with MB and LA at both N levels for all varieties, showed by similar ranking patterns across the three traits (**Table 5**).

**Table 5:** Comparative performance of 15 wheat varieties at vegetative stage. Data are means of fresh shoot biomass, leaf area at 49 DAS (n = 6) and estimated biomass, top-view area at 54 DAS (n = 9) under low and optimum N levels. Varieties are ranked in descending order of fresh biomass accumulation at low N level. In a column: dark green cells, the highest values; dark red cells, the lowest values. MB, measured fresh shoot biomass; LA, measured leaf area; EB, estimated shoot biomass; TVA, top-view area; n = sample number.

Variety	MB (g pot^-1^)				LA (cm^2^ pot^-1^)				EB (kPix pot^-1^)				TVA (kPix pot^-1^)			
	Low N		Optimum N		Low N		Optimum N		Low N		Optimum N		Low N		Optimum N	
Bobwhite	8.33		25.52		191.01		699.92		245.86		759.47		57.22		215.81	
Excalibur	8.04		36.63		238.46		1162.61		347.36		1059.88		74.14		296.44	
Yitpi	7.66		25.94		217.56		693.79		254.35		751.63		48.70		166.91	
Alsen	7.66		31.61		214.09		906.27		295.29		921.56		66.91		249.65	
Kukri	7.63		29.21		191.82		768.57		251.00		859.99		51.20		223.55	
RAC875	7.49		26.79		178.45		636.31		207.91		699.55		39.89		180.93	
Wyalkatchem	6.67		28.38		190.34		792.61		242.24		711.04		46.49		169.47	
Chara	6.59		21.97		174.86		599.51		218.43		663.29		39.50		153.32	
Baxter	6.29		25.97		173.00		784.56		233.44		840.74		49.58		218.80	
Pastor	6.10		32.06		157.68		831.87		251.98		849.85		51.37		224.07	
Kennedy	6.00		27.37		136.43		723.73		170.16		721.79		33.02		174.04	
Gladius	5.87		23.14		142.66		587.06		199.04		647.07		39.25		163.88	
Westonia	5.62		24.99		125.66		569.03		221.09		656.73		45.03		162.15	
Volcani DDI	5.49		19.68		123.79		413.69		199.42		614.73		43.58		163.12	
Drysdale	5.01		25.74		96.90		635.07		173.51		712.40		33.09		181.50	
ANOVA	N	V		N x V	N	V		N x V	N	V		N x V	N	V		N x V
s.e.d	0.083	1.052		1.440	4.76	40.27		52.22	13.30	25.00		36.65	5.59	7.15		11.25
p	<0.001	<0.001		0.003	<0.001	<0.001		<0.001	<0.001	<0.001		<0.001	<0.001	<0.001		<0.001
l.s.d (*p* = 0.05)	4.17	4.13			113.05	116.66			73.42	70.82			22.75	20.25		

**Figure 7:**
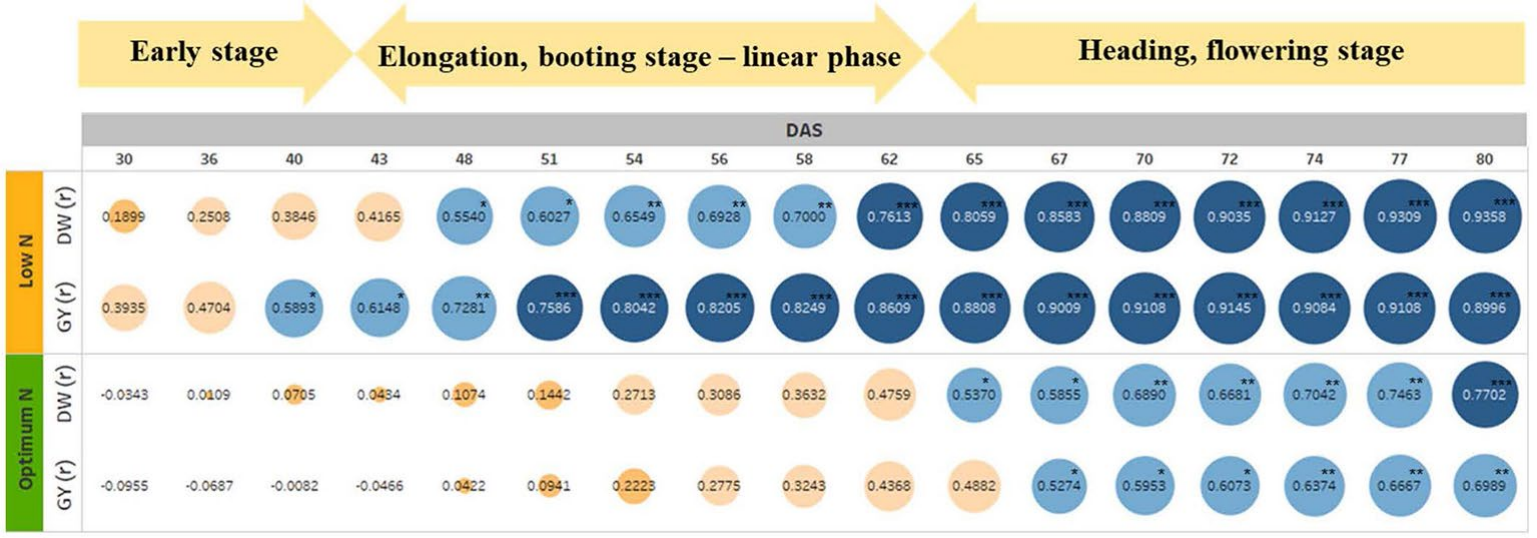
The relationship between estimated shoot biomass at different growth stages with harvest dry biomass and grain yield. The numbers inside the circle are correlation coefficients (r). Colour and circle size indicate r magnitude. DW, plant dry biomass per pot; GY, grain yield per pot. The asterisks are the statistically significant levels (* p ≤ 0.05; ** p ≤ 0.01; *** p ≤ 0.001).

To test if the estimated parameters at the vegetative stage could predict biomass accumulation and grain yield at harvest, we determined the association between EB on different DAS with final DW and GY (**Figure 7**). The heat map shows increasing positive correlations between EB and DW or GY at low N level from 30 DAS onwards, with the correlation peaking at 80 DAS coinciding with the start of the flowering period (**Figure 7** and **Supplementary Figure 1**). In contrast, the positive correlation between these parameters at optimum N only started at 48 DAS, also peaking at 80 DAS, even though the correlations were much lower compared to those under low N (**Figure 7**). Data also showed that EB observed at 60 DAS could possibly explain 50% and 70% of the variations in DW and GY at low N, respectively. Whereas, these figures were only approximately 18% and 14% at optimum N (**Figure 7**).

### Comparative Performance of Wheat Varieties Under Controlled and Field Conditions

To determine if better performing varieties in the greenhouse perform well in the field, we analysed the correlation between DW and GY of wheat plants grown under greenhouse and field conditions. We compared the performance of the 15 wheat varieties from this greenhouse trial to the same varieties grown in the field at Horsham, Victoria, Australia in 2013 (Nguyen et al., 2016). There were several correlations between DW and GY in the greenhouse and the field, with some trends identifiable (**Supplementary Table 2**). These results showed low to moderate positive correlation for DW, especially the DW of wheat plants in the greenhouse at both N levels was significantly correlated with that of field plants at 80 N, the N level gives optimum NUE (**Supplementary Table 2**). The GY at low N in the greenhouse and the three field N levels also demonstrated a positive correlation trend with each other (**Supplementary Table 2**) (Nguyen et al., 2016).

## Discussion

### An Advanced Phenotypic Screening Method for NUE Improvement in Wheat Under Controlled Environments

This work describes the development of a robust, high-throughput, reliable plant phenotyping method using automated digital imaging technology, that can be used to effectively screen a diverse range of wheat germplasm for NUE improvement at the vegetative stage in a controlled environment. The development of N-efficient wheat varieties through molecular breeding will undoubtedly contribute to the more effective use of N fertilizer, which is currently causing significant production and environmental costs (Cormier et al., 2016). However, NUE is a multi-genic trait and the lack of reliable phenotyping methods is currently a rate determining factor in NUE genetic improvement programs. These methods will aid in effectively screening breeding populations, phenotyping training populations for genomic selection, and evaluating breeding lines (Cabrera‐Bosquet et al., 2012; Araus et al., 2018; Campbell et al., 2018). Thus, the availability of efficient phenotyping methods that are capable of characterising and quantifying multiple NUE traits, will provide useful tools to wheat breeders. In the present study, our results have demonstrated an applicable and reproducible wheat growth assessment system in a controlled environment for NUE studies. The results also revealed corresponding performance between wheat varieties screened by the digital RGB imaging unit of automated plant phenotyping platform, compared to field conditions for NUE traits.

An optimal plant growth system that can precisely apply and manipulate nutrient supplies in a timely way will play an important role in N studies. Several potting systems using pre-fertilized mixes have been reported to screen wheat for improved NUE in greenhouse studies (Tian et al., 2015; Malik et al., 2016; Veres et al., 2017). Although effective, these systems pose challenges such as the ability to timely adjust the amount of N supply for a range of varieties with unknown and diverse N responsiveness. The gradually supplied liquid fertilizer method used here, has advantages over pre-fertilized potting mixes, especially when used in conjunction with an automated watering system, which can also accurately dispense a set volume of fertilizer solution (Nguyen et al., 2018). Since water highly interacts with N availability in the growth media (Nguyen et al., 2017), this system will ensure an adequate supply of water for plants so that NUtE is not affected by either a shortage or excess of water, and will help reduce the time and labour costs associated with manual watering. More importantly, it was demonstrated that the current screening method could effectively produce significant variations in NUE traits (NUEb and NUEg) among wheat varieties, which will facilitate selection in screening processes.

### Vegetative Screens by RGB Imaging for N-Efficient Wheat Genotypes

Early vigour, biomass accumulation, grain yield and grain protein are key criteria for the selection of N-efficient wheat materials (Nguyen and Kant, 2018). Since wheat grain yield is largely determined by the availability of carbohydrate reserves in the leaves and stems pre-anthesis, the higher the biomass accumulation during vegetative growth, the higher DW and GY at harvest (Reynolds et al., 2009; Li et al., 2013). Improving wheat yield potential by increasing DW at harvest has been demonstrated as an achievable and feasible strategy in breeding programs (Aparicio et al., 2002; Hawkesford, 2017). The primary objective of vegetative screens is to save time and costs, while still being able to effectively identify wheat genotypes which perform better for DW and GY at harvest in controlled environments and the field; ultimately speeding up breeding outcomes (Aparicio et al., 2000; Aparicio et al., 2002). In the present study, results confirmed that the automated RGB imaging unit was effective in estimating biomass accumulation from early vegetative to heading stages with a high degree of accuracy. The high correlations between EB and TVA and important traits such as MB and LA confirmed that the formers can be used as surrogates of the latter to evaluate the performance of wheat varieties for NUE at vegetative stage without destructive samplings. Our results also demonstrated that the performance of wheat varieties could be assessed effectively at early vegetative stages, as plant status during the linear growth phase truly reflects the potential at maturity regarding DW and GY. Being able to phenotype traits early in a high-throughput, precise and reproducible manner is a clear advantage in greenhouse screens and crucial for accelerating the breeding of improved NUE varieties (Ly et al., 2017). Vegetative screens, using conventional phenotyping methods, have also been successfully used for quantitative trait loci (QTL) mapping of early growth traits such as seedling height or shoot biomass for improved NUE in wheat (An et al., 2006; Guo et al., 2012). Highly accurate estimates of vegetation coverage of field grown wheat at booting stage using digital RGB images have been reported previously (Lukina et al., 1999). Moreover, the automated RGB imaging unit used in this study, was also advantageous against other proximal sensing tools, since it was not negatively influenced by genotypic variations or N levels observed elsewhere (Babar et al., 2006; Nguyen et al., 2016).

However, the causal relationship between vegetative performance and both DW and GY isn’t fully understood. Under low N conditions, the EB of wheat plants was better correlated with GY than DW at early growth (40 DAS) and with correlation strength increasing until 74 DAS. Whereas, the correlation started at 67 DAS under optimum N conditions. In wheat, the remobilization of N reserved in vegetative parts, such as shoots and roots, before flowering, contributes up to 95% of grain N content at maturity (Palta and Fillery, 1995). Previous studies suggested that higher grain yield and grain NUtE in wheat were determined by a higher N remobilization efficiency, which was subject to genotypic assimilation efficiency and the availability of stored N in vegetative parts of the plants (Barbottin et al., 2005; Tian et al., 2015). Additionally, the remobilization of reserved carbohydrate pre-anthesis in wheat contributes up to 20% of grain yield under favourable conditions and up to 60% under stressful conditions, including N stress (Li et al., 2013), which was supported by our WSC assay results. Since NUE-related traits, e.g. remobilization efficiency, were highly expressed under low N conditions (Barbottin et al., 2005; Lammerts Van Bueren and Struik, 2017), it is likely that most of the reserved N and WSC in the wheat plants were translocated to grain yield before maturity due to N stress, leading to higher correlations between vegetative EB, DW and GY. Whereas, abundant N supplies meant that a large portion of the N and WSC reserved in shoots and roots were leftover in the DW, resulting in a lower correlation between EB from early stages and DW and GY (Barbottin et al., 2005; Gaju et al., 2014). Thus, selection might focus on the performance of wheat genotypes under low N rather than optimum N conditions for vegetative screens. The small variations in NUEg within N levels among varieties again supports this hypothesis. Based on growth analysis in the current study of 15 wheat varieties, it is recommended that EB collected at 60 DAS, that coincides with booting stages, can be used to compare vegetative performance of wheat varieties.

### Perspective of Image-Based Phenotyping for NUE Improvement in Wheat Under Field Conditions

One of the biggest challenges in the development of N-efficient wheat varieties is necessity of developing an effective screening system in controlled environments that can effectively foresee the performance of wheat varieties under field conditions (Nguyen and Kant, 2018). In the present study, we compared the performance of wheat varieties for NUE under controlled and field conditions. We observed moderate and low-level correlations between greenhouse and field data for the DW and GY of identical wheat varieties, respectively (**Supplementary Table 2**). Interestingly, the DW of wheat varieties appears more consistent under both N levels in greenhouse compared to the 80 N, the optimum level under field conditions (**Supplementary Table 2**). The inconsistent performance of varieties under greenhouse and field has been well documented (Poorter et al., 2012; Junker et al., 2015). Quite often, genotypes selected from the controlled environments do not substantiate their performance under field conditions, because of significant competition among plants within plots under field conditions, which is not present for individual plants in pots in controlled environments (Araus and Cairns, 2014; Fischer and Rebetzke, 2018). Associations between greenhouse and field trials using the same varieties is further complicated by other environmental factors such as soil type, microorganisms, N and water availability (Cormier et al., 2016; Nguyen et al., 2017). However, several studies have reported a causal relationship between greenhouse screens and the field performance of crops (Chapuis et al., 2012; Pardo et al., 2015; Peirone et al., 2018). Therefore, results from vegetative screens in greenhouses, like those in the current study, can still be useful indicators of the performance of genotypes for NUE, which can help reduce the time and cost of developing new breeding materials.

Non-invasive remote sensing and imaging, using sensors and cameras, has been successfully applied to field crop phenotyping for NUE improvement (Nguyen and Kant, 2018). Simple to set up and cost effective conventional digital cameras have been effectively used as assessment tools for leaf area index and biomass in cereals (Casadesús and Villegas, 2014). The advent of various ground-based and aerial-based plant phenotyping platforms has made the estimation of final biomass and grain yield in wheat faster, more accurate and economical (Reyniers et al., 2006; Wang et al., 2014; Kefauver et al., 2017). Vegetation indices have been used to estimate biomass and grain yield under varying N supplies with high accuracy (Serrano et al., 2000). However, all the above-mentioned phenotyping platforms were deployed at the booting and heading stages to predict yield and final biomass, since biomass accumulation peaks at anthesis (Aparicio et al., 2000; Chang et al., 2005; Malhi et al., 2006); but, none of them were designed to predict final biomass and grain yield at early vegetative stages. Early vegetative prediction of N-efficient genotypes by high-throughput phenotyping will be especially useful, particularly when applied with genomic selection for NUE (Ly et al., 2017). This is particularly helpful for sensor and image based phenotyping in the field because vegetation indices will possibly become saturated if the leaf area index of the canopy is > 3 (Aparicio et al., 2000; Serrano et al., 2000). Several recent reports showed the potential of RGB imaging technology to study early crop growth and yield for NUE improvement in the field. Prey et al. (2018) used canopy cover from RGB imaging as a criterion to assess early vigour in wheat. In a similar approach, Buchaillot et al. (2019) used vegetation indices generated by both ground and aerial based RGB sensors at the vegetative stage in combination with crop’s agronomic parameters, to successfully develop regression models for yield prediction of maize genotypes. Since TVA, observed here, was highly correlated with other traits, an avenue for further investigation is the deployment of digital RGB cameras, either handheld or mounted on ground or aerial vehicles (Araus and Kefauver, 2018; Fernandez-Gallego et al., 2019; Gracia-Romero et al., 2019) to capture and assess the performance of wheat genotypes for NUE traits at the linear growth phase under field conditions.

## Conclusions

Here, we have described the development of a robust, high-throughput and reliable screening method at vegetative growth phases to investigate NUE improvements in wheat under controlled environment. Our results have shown that this digital RGB imaging method is strongly correlated to important NUE traits such as MB of wheat varieties. The observed relationship between controlled and field conditions for the same varieties indicates that greenhouse screening could be used to prioritise germplasm for subsequent field studies. Therefore, the application of this designated wheat growth system in conjunction with the digital imaging will provide breeders with an excellent assessment tool to enable the rapid phenotyping of diverse wheat genotypes to select N-efficient germplasm. This screening method may also provide a basis for the rapid phenotyping method of other crop species to identify germplasm efficient to a range of nutrients and stresses.

## Data Availability Statement

All datasets generated for this study are included in the manuscript, except raw RGB images which are available upon request.

## Author Contributions

SK and GN designed the experiments. GN, PM, LM, JV, and ET-K conducted the experiment. GN performed data curation and statistical analysis and wrote the first draft of the manuscript. SK, ET-K, PM, LM, JV, and GN contributed to manuscript editing and revision, and read and approved the submitted version.

## Conflict of Interest

The authors declare that the research was conducted in the absence of any commercial or financial relationships that could be construed as a potential conflict of interest.

## Supplementary Material

Supplementary Figure 1 Phenology of wheat varieties under two N levels. **(A)** days from sowing to heading; **(B)** days from sowing to physiological maturity. Bars are mean of nine replicates and capped lines are standard errors.
