# Supplementary figures and images for: A Robust Automated Image-Based Phenotyping Method for Rapid Vegetative Screening of Wheat Germplasm for Nitrogen Use Efficiency
Source: Front Plant Sci. 2019 Nov 5;10:1372. doi: 10.3389/fpls.2019.01372 (PMC6849468; doi:10.3389/fpls.2019.01372)

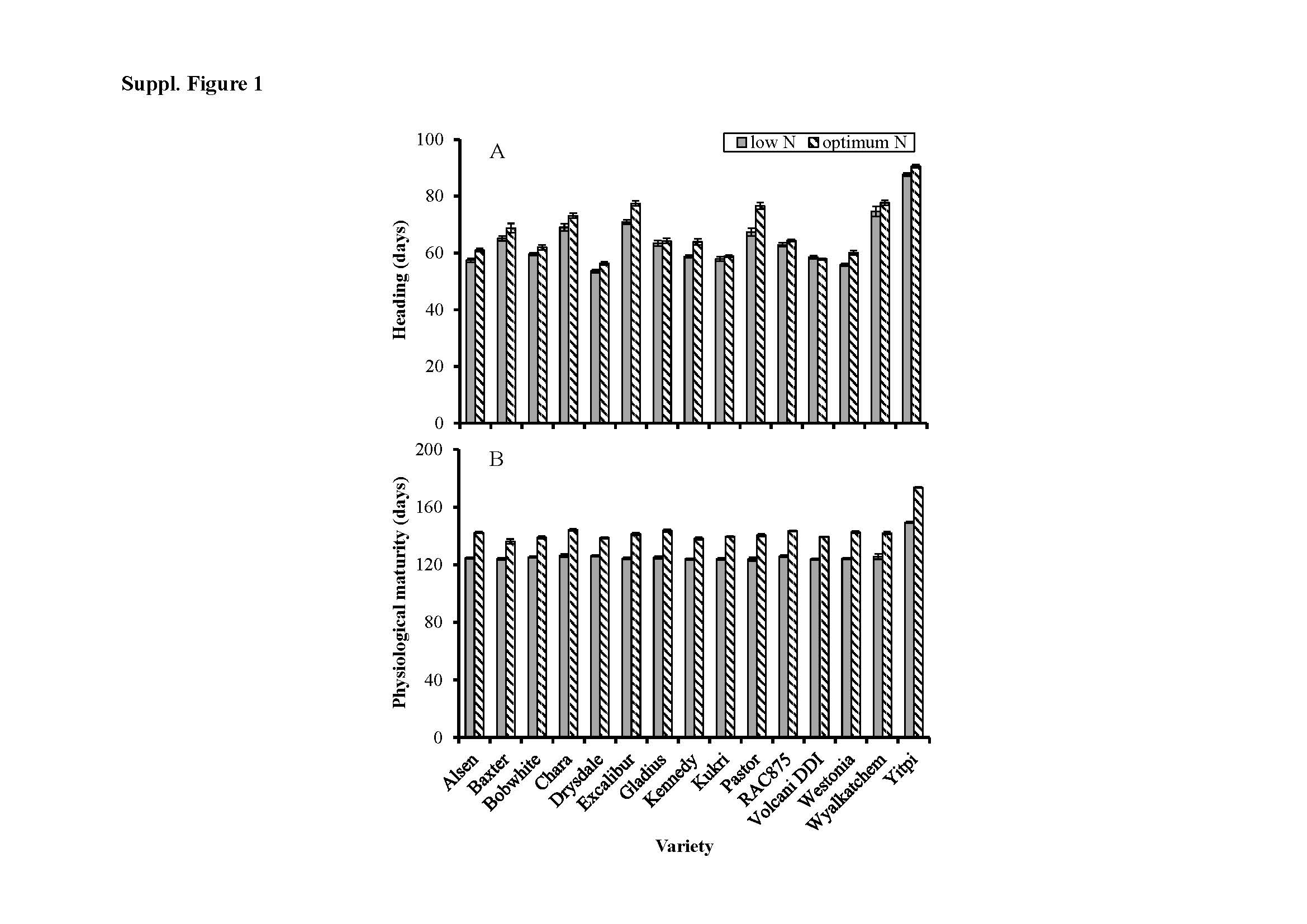

Supplement: Supplementary Figure 1 — Phenology of wheat varieties under two N levels. (A) days from sowing to heading; (B) days from sowing to physiological maturity. Bars are mean of nine replicates and capped lines are standard errors. [file Image_1.jpeg]
