# Supplementary material for: A Robust Automated Image-Based Phenotyping Method for Rapid Vegetative Screening of Wheat Germplasm for Nitrogen Use Efficiency
Source: Front Plant Sci. 2019 Nov 5;10:1372. doi: 10.3389/fpls.2019.01372 (PMC6849468; doi:10.3389/fpls.2019.01372)
Supplement: Supplementary file 2 [file DataSheet_1.docx]

**Suppl. Table 1:** Component of the nutrient solutions used in experiments


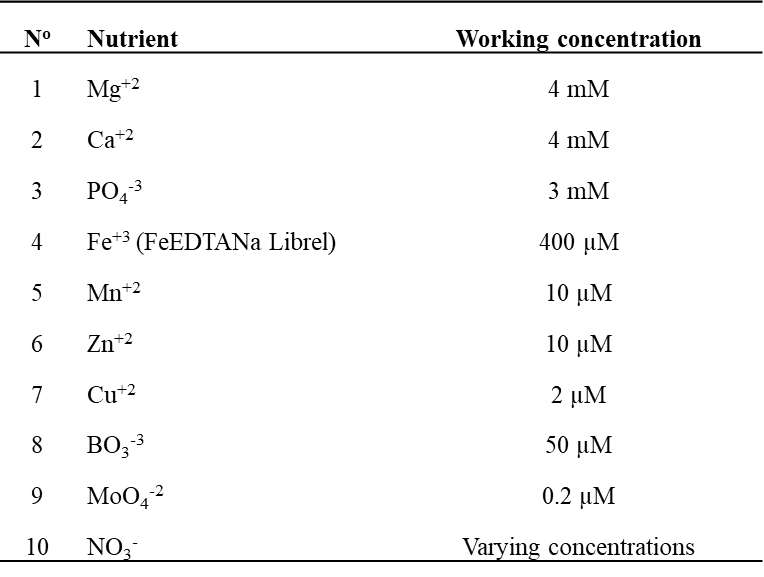


**Suppl. Table 2:** Coefficients (r) of the correlation between shoot dry biomass and grain yield of 15 wheat varieties at harvest under greenhouse and field conditions. DW, shoot dry biomass; GY, grain yield. Field data were adopted from Nguyen et al. (2016). Correlation coefficients marked with an asterisk are statistically significant at 5%.

**
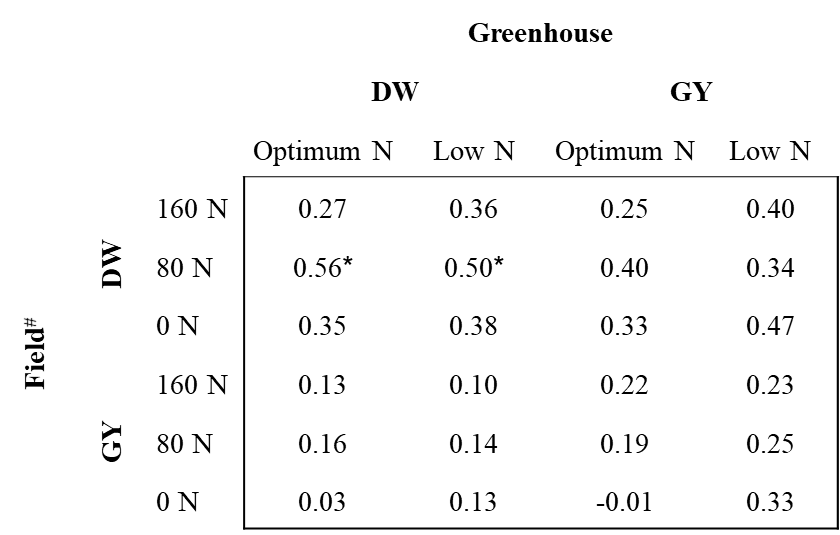
**

^#^Field data were adopted from Nguyen et al. (2016)

^#^Field data were adopted from Nguyen *et al.,* (2016)
